# Platelet, Antiplatelet Therapy and Metabolic Dysfunction-Associated Steatotic Liver Disease: A Narrative Review

**DOI:** 10.3390/life14040473

**Published:** 2024-04-04

**Authors:** Andrea Boccatonda, Lorenza Del Cane, Lara Marola, Damiano D’Ardes, Gianfranco Lessiani, Nicoletta di Gregorio, Claudio Ferri, Francesco Cipollone, Carla Serra, Francesca Santilli, Fabio Piscaglia

**Affiliations:** 1 Internal Medicine, Bentivoglio Hospital, AUSL Bologna, 40010 Bentivoglio, Italy; 2 Department of Medical and Surgical Sciences, University of Bologna, 40138 Bologna, Italy; fabio.piscaglia@unibo.it; 3 Nephrology Unit, Department of Life, Health & Environmental Sciences and Internal Medicine, University of L’Aquila, ASL Avezzano-Sulmona-L’Aquila, San Salvatore Hospital, 67100 L’Aquila, Italy; lory_ok@msn.com (L.D.C.); lara_marola@hotmail.it (L.M.); nicolettadigregorio@yahoo.it (N.d.G.); claudio.ferri@univaqcc.it (C.F.); 4 Institute of “Clinica Medica”, Department of Medicine and Aging Science, “G. D’Annunzio” University of Chieti, 66100 Chieti, Italyfrancesco.cipollone@unich.it (F.C.); 5 Villa Serena Hospital, 65030 Città Sant’Angelo, Italy; gf.lessiani@gmail.com; 6 Interventional, Diagnostic and Therapeutic Ultrasound Unit, IRCCS, Azienda Ospedaliero-Universitaria di Bologna, 40138 Bologna, Italy; carla.serra@aosp.bo.it; 7 Department of Medicine and Aging Sciences, Center for Advanced Studies and Technology, University of Chieti, 66100 Chieti, Italy; francesca.santilli@unich.it; 8 Division of Internal Medicine, Hepatobiliary and Immunoallergic Diseases, IRCCS Azienda Ospedaliero-Universitaria di Bologna, 40138 Bologna, Italy

**Keywords:** cancer, NAFLD, MASLD, aspirin, thromboxane

## Abstract

Metabolic dysfunction-associated steatotic liver disease (MASLD) is not only related to traditional cardiovascular risk factors like type 2 diabetes mellitus and obesity, but it is also an independent risk factor for the development of cardiovascular disease. MASLD has been shown to be independently related to endothelial dysfunction and atherosclerosis. MASLD is characterized by a chronic proinflammatory response that, in turn, may induce a prothrombotic state. Several mechanisms such as endothelial and platelet dysfunction, changes in the coagulative factors, lower fibrinolytic activity can contribute to induce the prothrombotic state. Platelets are players and addresses of metabolic dysregulation; obesity and insulin resistance are related to platelet hyperactivation. Furthermore, platelets can exert a direct effect on liver cells, particularly through the release of mediators from granules. Growing data in literature support the use of antiplatelet agent as a treatment for MASLD. The use of antiplatelets drugs seems to exert beneficial effects on hepatocellular carcinoma prevention in patients with MASLD, since platelets contribute to fibrosis progression and cancer development. This review aims to summarize the main data on the role of platelets in the pathogenesis of MASLD and its main complications such as cardiovascular events and the development of liver fibrosis. Furthermore, we will examine the role of antiplatelet therapy not only in the prevention and treatment of cardiovascular events but also as a possible anti-fibrotic and anti-tumor agent.

## 1. Introduction

Non-alcoholic fatty liver disease (NAFLD) represents the most common cause of liver disease worldwide [1]. Recently, some guidelines have moved towards changing the terminology. The new nomenclature for a metabolic-associated fatty liver disease (MAFLD) was therefore suggested in 2020 to overcome the concerns correlated with the old terminology [2]. MAFLD diagnosis is based on the presence of liver steatosis and at least one of the following three conditions: type 2 diabetes mellitus (T2DM), obesity, or metabolic dysregulation [2]. A multi-society Delphi consensus statement in 2023 proposed a novel nomenclature about steatotic liver disease (SLD) [3]. SLD can be detected histologically or by imaging, and it displays many potential reasons [3]. Metabolic dysfunction-associated SLD (MASLD) is defined as the presence of liver steatosis along with one cardiometabolic risk factor and no other discernible cause [3]. Individuals with MASLD and steatohepatitis will be defined as metabolic dysfunction-associated steatohepatitis (MASH) [3].

MASLD has been shown to be related not only to traditional cardiovascular risk factors like T2DM [4] and obesity [5], but it is also an independent risk factor for the development of cardiovascular disease [6,7]. 

NAFLD has been shown to be independently related to endothelial dysfunction and atherosclerosis [8]. NAFLD is characterized by a chronic proinflammatory response that, in turn, may induce a prothrombotic state [9]. Several mechanisms such as endothelial and platelet dysfunction, changes in the coagulative molecules, and lower fibrinolytic activity can contribute to induce the prothrombotic state [10]. The “thrombophilic” state correlated with liver diseases may induce macrovascular and microvascular events; in particular, the formation of microthrombi in the hepatic venules can affect the course of liver disease [11]. The process of hepatic injury induced by the microthrombi in cirrhotic patients is called “parenchymal extinction” [12,13]. The occlusion of liver and portal venules by microthrombi induce the disruption of the normal blood flow, thus leading to congestion, local ischemia, and parenchymal injury [12,13]. The subsequent hepatocyte apoptosis induces the parenchymal damage, which is replaced by fibrous septa [12]. 

Moreover, hepatic stellate cells can be activated directly by increased amounts of thrombin and coagulative molecules, even without intrahepatic thrombosis [11,13]. Furthermore, a growing body of evidence demonstrated that antiplatelet treatment not only prevented atherothrombotic events in subjects affected by liver diseases, but it could also potentially reduce liver disease progression [14].

This review aims to summarize the role of platelets in the pathophysiology of patients with NAFLD and its complications, and to examine the evidence on the use of antiplatelet therapy in this category of patients.

## 2. Coagulation Cascade in Non-Alcoholic Fatty Liver Disease

There are currently conflicting data on the dysregulation of the coagulation cascade in subjects with NAFLD, especially in the early stages. Kotronen et al. performed a study on 54 subjects with NAFLD demonstrating that higher levels of coagulation factors VIII, XI, and XII were related to the hepatic fat amount [15]. Another study on 273 subjects with histologically proved NAFLD showed high amounts of fibrinogen, factor VIII, and von Willebrand factor (vWF) factor and lower antithrombin levels than in healthy subjects [16]. Otherwise, the levels of coagulation factors were not related to histological changes, and the PAI-1 levels were the only ones related to MAFLD degree [16]. Tripodi et al. demonstrated a significant prothrombotic state in 113 subjects with different stages of NAFLD, that was more evident in the subjects with advanced liver disease (cirrhosis) than in those with simple steatosis [17]. Many other works in NAFLD/NASH subjects have demonstrated increased levels of factors VII, VIII, IX, XII, vWF, and tissue factor (TF), probably related to higher C-reactive protein (CRP), plasminogen activator inhibitor 1 (PAI-1), and fibrinogen levels [9,11,13,15]. Otherwise, two works performed on biopsy-proven NAFLD patients argued that the prothrombotic dysregulation was most probably correlated with obesity or insulin resistance than with hepatic fat amount [18,19]. Moreover, Assy et al. showed that several molecules in the coagulation cascade are early increased in MAFLD, but tend to decrease according to liver disease progression, thus leading to a higher risk of bleeding [20]. Eventually, the concept of “rebalanced hemostasis” is actually well established in advanced forms of the disease that have progressed to cirrhosis [21]. 

## 3. Role of Platelets in MASLD (NAFLD)

### 3.1. Platelets, Metabolism Dysregulation, and Liver Disease

**Figure 1:**
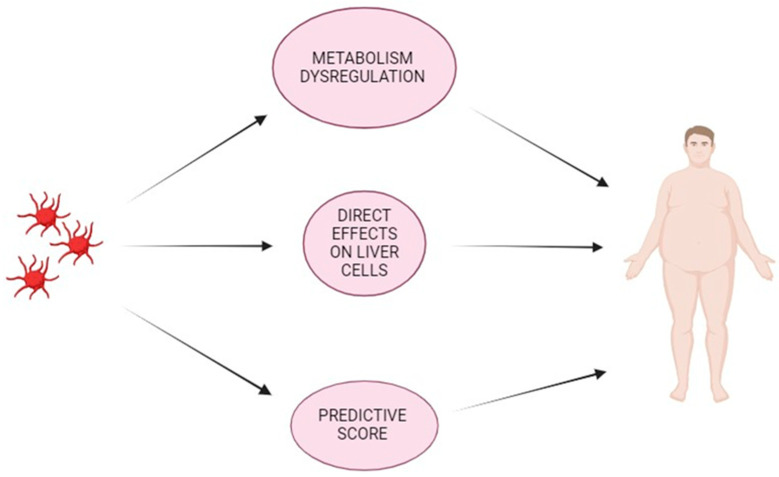
Summary figure on the role of platelets in patients with MASLD (NAFLD). Platelets are players and aggressors of metabolic dysregulation; obesity and insulin resistance are related to platelet hyperactivation. Furthermore, platelets can exert a direct effect on liver cells, particularly through the release of mediators from granules. Furthermore, platelets are included in the majority of clinical scores linked to liver disease and risk of evolution towards cirrhosis. Created in https://www.biorender.com/.

NAFLD/NASH often develops in the context of obesity, metabolic syndrome, and T2DM [22] (Figure 1). NAFLD/NASH induces a chronic inflammatory state of the liver, which is characterized by a complex pathophysiology. Lipid species can induce an inflammatory response in the liver by activating infiltrating and resident immune cells. There is a relevant correlation between hepatic fat amount and inflammatory mediators and oxidative stress molecules [23]. 

NAFLD patients are characterized by low levels of plasma endogenous secretory receptor for advanced glycation end products (esRAGE), interleukin (IL)-10, and adiponectin, and increased amounts of CD40 ligand, endogenous thrombin potential, and oxidized low-density lipoproteins (LDL) [24]. The RAGE axis is involved in several diseases, such as T2DM, atherothrombosis, chronic kidney disease, dementia, cancer, and aging [25,26]. Circulating soluble forms of RAGE, derived from receptor ectodomain shedding and splice variant esRAGE secretion, can oppose RAGE-mediated pathogenesis, by functioning as a decoy [25,26]. Many works have showed low levels of esRAGE to be a reliable biomarker of the ligand–RAGE pathway hyper activation and an inadequate endogenous protective response [25,26].

Liver fat is directly related to CRP, isoprostanes, IL-6, intercellular adhesion molecule-1 (ICAM-1), and P-selectin levels [23]. ICAM-1 and P-selectin levels are significantly increased in subjects with liver steatosis and elevated ALT in comparison with those without fatty liver [23,27]. In vitro studies have demonstrated that hepatocytes exposure to fatty acids can trigger the expression of tumor necrosis factor (TNF)-α [28], IL-6, ICAM-1, [29], and isoprostanes [30], through nuclear factor-κB (NF-kB) [31]. Chronic hepatic activation of the NF-κB pathway can induce IL-6-mediated insulin resistance; TNF-α inhibition decreases liver fatty acid oxidation and insulin resistance by Kupffer cell activation [32,33].

**Figure 2:**
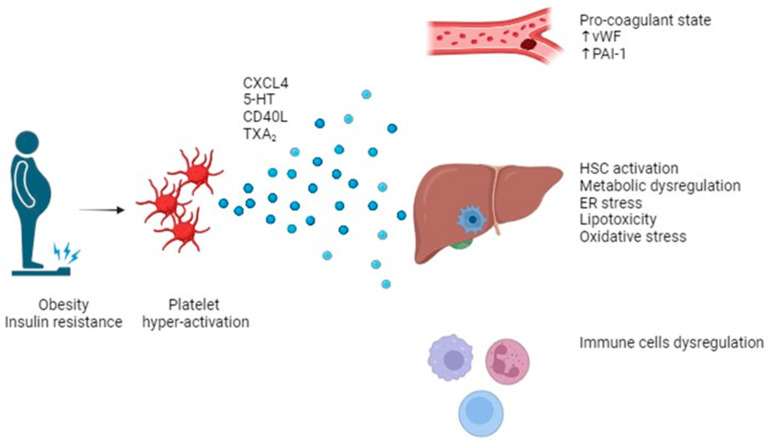
Obesity and insulin resistance are correlated with platelet hyper-activation. Upon activation, platelets release some molecules and mediators harbored in their granules such as 5-HT, CD40L, and TXA_2._ Those molecules are involved in determining a pro-thrombotic and pro-coagulant state. Moreover, many pathways are involved in direct liver damage by HSC activation, ER stress, lipotoxicity, and oxidative stress. Eventually, there is a cross-talking between platelets and immune cells, thus inducing a dysregulated immune response involved in liver damage and in determining coagulative disorder. Abbreviations: chemokine (C-X-C motif) ligand 4, CXCL4; 5-hydroxytryptamine, 5-HT; CD40 ligand, CD40L; thromboxane A_2_, TXA_2_; von Willebrand factor, vWF; plasminogen activator inhibitor 1, PAI-1; hepatic stem cell, HSC; endoplasmic reticulum, ER. Created in https://www.biorender.com/.

In obese subjects, platelets are characterized by increased aggregability and activation [34] (Figure 2). The adipokine leptin could represent a connection between platelets, obesity, and MAFLD. Leptin levels are related to NAFLD degree, and atherothrombotic events can be triggered in a platelet leptin receptor-dependent manner in those patients [35]. Indeed, leptin induces adenosine diphosphate (ADP)-related platelet aggregation at clinically relevant levels [36]. Thromboxane (TX)A_2_ levels and liver TXA_2_ receptor amounts are increased in MAFLD patients more than healthy subjects [37]. In obese and insulin resistant patients, there are higher plasmatic levels of P-selectin, which decrease after weight loss [38].

In obese patients, high levels of circulating platelet-derived microvesicles (PMVs) are directly related to BMI and waist circumference. In obese patients, PMVs display an heterogeneous size and distribution, with different amounts of molecules related to atherothrombosis and carcinogenesis [39]. Notably, weight reduction can decrease the circulating levels of PMVs [40]. PMVs may bear functional receptors from platelet membranes, thus exerting different effects. The exposure of phosphatidylserine is related to pro-thrombotic and inflammatory milieu [41]. PMVs can regulate cyclooxygenase-2 (COX-2) and prostacyclin (PGI_2_) levels in endothelial cells [42]. Moreover, PMVs are involved in interactions between monocytes and endothelial cells by regulating the expression of ICAM-1 [43], and the recruitment of neutrophils by P-Selectin and IL-1 expression [44]. Furthermore, they can induce the release of inflammatory molecules such as CD40-ligand (CD40L), IL-1, IL-6, and TNF-α [45], thus increasing the activation of the classic complement pathway [46]. 

Platelet hyper-activation is also observed in patients with hypercholesterolemia, together with higher expression of fibrinogen binding, P-selectin, superoxide anion, and enhanced TXA_2_ production. Plasma from patients with high cholesterol levels is characterized by increased amounts of platelet activation molecules, such as CD40L, soluble P-selectin, platelet factor 4 (PF-4), and thromboglobulin [47]. Notably, triglyceride-rich particles can directly induce platelet activation [48]. 

In patients with insulin resistance, the adipokines resistin, leptin, PAI-1, and retinol binding protein 4 (RBP4) can dysregulate the insulin receptor substrate-1 (IRS-1) level in megakaryocytes, thus disrupting insulin signaling in platelets [49,50]. High glucose levels in diabetes are related to platelet hyperactivation, enhanced fibrinogen binding, and TXA_2_ production [51,52,53]. In obese and insulin-resistant subjects, platelets demonstrated a dysregulated response to nitric oxide (NO) and changed the cyclin guanosine monophosphate (cGMP)-dependent protein kinase (PKG) signaling system. 

Moreover, the inhibition of platelet activation by PGI_2_ and the activation of the cyclin adenosine monophosphate (cAMP)-dependent protein-kinase (PKA) pathways are dysregulated [54]. In that context, platelet activation signals are overexpressed such as the higher free intracellular calcium amount and the release of platelet activation molecules such as PMVs [52,55]. 

High glucose levels are correlated with the release of pro-oxidant molecules, which can enhance cytosolic phospholipase A2 signaling, thus catalyzing arachidonic acid release and TXA_2_ generation. Aldose reductase pathway activation can enhance TXA_2_ biosynthesis amplified by exposure to collagen [52]. 

In diabetes, TXA_2_-mediated platelet hyper-activation is driven by the protein kinase C (PKC)/p38 mitogen activated protein kinase (MAPK) pathway, and it also related to increased CD40L release [52,56]. CD40L is a member of the TNF superfamily, and it is more expressed in the MAFLD platelet surface [57]. In animal studies on insulin resistance, the genetic or antibody-mediated disruption of CD40L signaling have been shown to counteract the diet effects on fat liver amount, adipose tissue increase, and insulin resistance, by acting on liver very low-density lipoprotein (VLDL) release and genes adjusting lipid metabolism [58].

Moreover, diabetes is related to damage and loss of function of mitochondria in platelets, cytochrome c release, and caspase-3 activation, thus inducing platelet apoptosis [59]. 

### 3.2. Platelet & Liver

A growing body of evidence has demonstrated that platelets play an active and direct role in the pathogenesis of liver disease and inflammation. Platelet hyperactivation plays a main role in cytotoxic T lymphocyte (CTL)-mediated hepatic cell damage in viral hepatitis [60]. Platelets can be recruited by Kupffer cells to the liver in early and late phases of NAFLD and NASH [61]. In the early stages, this process involves hyaluronan and platelet CD44 [61]. Glycoprotein Ib platelet subunit alpha (GPIbα) is a platelet surface membrane glycoprotein, and it plays a role in the interaction between platelets and Kupffer cells at late but not at early stages of fibrosis development [61]. Furthermore, there are no clear data about the role of platelet GPIIb/IIIa in steatohepatitis [61]. Once recruited at the liver level, platelets can release granules containing several molecules [61]. Some pro-aggregatory molecules such as ADP, serotonin, and thrombin along with inflammatory cytokines, chemokines, and growth factors can be released from α and δ granules in the microenvironment [62,63]. Platelets can store and produce IL-1, PAI-1, and tissue factor (TF). Those molecules released from platelets can dysregulate gene expression in endothelial cells, leukocytes, stromal cells, and fibroblasts, thereby directly contributing to an enhanced inflammatory response [64]. In animal models and human subjects affected by MAFLD, there are increased blood amounts of molecules harbored in granules from platelets. Thrombospondin (TSP-1) is a molecule present in platelets, but it is also synthesized by liver stellate cells, Kupffer cells, endothelial cells, and adipocytes, and it can exert a positive effect on MAFLD by counteracting the expression of genes favoring lipid generation [65].

Malehmir et al. demonstrated that anti-GPIbα antibody treatment can decrease some cytokines and platelet-derived molecules, thus arguing that molecules contained in α granules yield an increase in immune cell attracting chemokines/cytokines [61]. The same authors argued that three main ligands of GPIbα (vWF, macrophage integrin-1, and P-selectin) are not relevant for NASH, but they reinforced the concept of the pro-inflammatory effect of α-granules in liver immune cell recruitment [61]. Furthermore, selectins are involved in immune cell recruitment to hepatic microvasculature upon an inflammatory response [66]. Eventually, GPIbα can play a role in disease development independent of a ligand [67]. Notably, anti-GPIbα antibody treatment can exert a therapeutical anti-NASH effect [61,68,69]. 

Indeed, both ticagrelor and anti-GPIbα antibody treatment can partially revert fibrosis on liver [61]. Therefore, P2Y12 antagonist treatment, depletion of functional GPIbα, or lack of α-granules decrease activation, accumulation, and adhesion of platelets to the liver endothelium, and they may also decrease immune-cell recruitment to the liver, thus exerting beneficial effects against liver damage and fibrosis development [61]. 

In models of liver disease, platelets have been shown to regulate gene expression in hepatocytes and deliver genetic signals to target cells. An in vitro study with hepatoblastoma cell line (HepG2) demonstrated the direct transfer of mRNA from platelets to hepatocytes by internalizing platelets. Platelets internalization has been also demonstrated in an animal model after a partial hepatectomy, and it was related to hepatocyte proliferation. On the other hand, enzymatic removal of platelet-derived ribonucleic acid (RNA) decreased hepatocyte proliferation [70]. Moreover, micro-RNA (miRNA) could be transferred from platelets to hepatocytes through the release of microparticles. PMP bearing miR-25-3p induced hepatocyte proliferation by changing gene expression [71]. 

Platelets may cross-talk with hepatic stellate cells by some molecules with pro- and anti-fibrotic effects [72]. Adenine nucleotides and hepatocyte growth factor contained in platelet granules display antifibrotic effects [73], and those effects are proved by the decrease in liver fibrosis by platelet-rich plasma (PRP) treatment [74].

Activated platelets exert a profibrotic effect by inducing hepatic micro-thrombosis [75] and through TGF [76], platelet-derived growth factor subunit B (PDGF-B) [77], vWF [78], and platelet-derived sphingosine-1-phosphate signaling [79] which increase collagen secretion by hepatic stellate cells [80], thus becoming myofibroblasts [81]. 

The role of platelets to liver inflammation was highlighted by immunohistochemical staining on liver biopsy specimens, which demonstrated the presence of great amounts of platelet and neutrophil extracellular traps (NETs) in the liver, with a relation to NAFLD activity score. Circulating platelets from MAFLD patients were characterized by a relevant increase in inflammatory transcripts, while leukocytes were not [82].

## 4. Platelet and Predictive Score of Liver Fibrosis

NAFLD patients are characterized by higher values of platelet distribution width (PDW) compared to healthy subjects [83,84]. Larger platelets display higher amounts of granules and adhesion receptors, thus leading to increased platelet activation [84]. PDW is directly correlated with platelet size, thus reflecting the heterogeneity in platelet morphology; this changes upon platelet activation [83]. Cao et al. demonstrated platelet count and PDW to be inversely related to fibrosis stage [85]. 

Moreover, lower platelet count and higher mean platelet volume (MPV) have been shown to be independent NAFLD predictors [86]. Several previous studies reported that steatosis was related to an increase in MPV values [86,87]. A recent Korean study showed a relevant correlation between MASLD and higher MPV values in 628 obese subjects [87]. A recent meta-analysis analyzing findings from eight works confirmed that MASLD patients had significantly increased MPV values compared to healthy controls [88]. Another work performed on 100 patients with biopsy-proven NAFLD demonstrated a significant stepwise increase in MPV levels from subjects with normal histology through patients with simple steatosis to those with steatohepatitis; MPV was significantly related to the histological features of steatohepatitis, such as steatosis, inflammation, ballooning, and fibrosis [89]. Those observations can be explained by the fact that an increased systemic inflammatory response with the release of cytokines induced by NAFLD can change the platelet size, and larger platelets can release more granules and prothrombotic molecules [88,90].

The platelet count is based on the balance between the production and destruction of platelets. In chronic liver diseases, the increase in spleen volume induces the sequestration and the destruction of platelets [91]. In NAFLD patients, platelet count was negatively related to the degree and the severity of liver fibrosis [92,93,94,95]. Indeed, fibrosis arises around the central veins, and portal hypertension seems to be primarily the result of central vein occlusion in NAFLD progression to NASH [96]. Moreover, platelets play a role in liver fibrosis by lowering the expression of TGF-β and by enhancing the expression of matrix metalloproteinases [95]. 

Shah et al. investigated the diagnostic accuracy of different scoring systems in 541 NAFLD patients [97]. The area under the receiver operating characteristic (AUROC) of the fibrosis index based on the 4 Factors (FIB4) index, NAFLD fibrosis score, the aspartate aminotransferase to platelet ratio index (APRI), and the AST-to-platelet ratio was used to evaluate the diagnostic performance for hepatic fibrosis stages ≥ stage 3; these were 0.802, 0.768, 0.730, and 0.720, respectively [97]. The AUROC of the platelet count to detect NAFLD patients with hepatic fibrosis ≥ stage 3 was 0.774 [92]. Indeed, the platelet count shows almost the same diagnostic accuracy as other biomarkers of liver fibrosis [92]. Therefore, it would be interesting to establish a threshold or range of platelet count that could suggest evolution towards hepatic fibrosis in patients with NAFLD.

In the work of Yoneda et al., the most accurate platelet count threshold to detect advanced fibrosis (stage 3–4) was 19.2 × 10^4^/μL, and that for cirrhosis diagnosis (stage 4) was 15.3 × 10^4^/μL in patients with NAFLD [92]. Therefore, MASLD patients with platelet counts ≤ 19.2 × 10^4^/μL should be monitored, because it is likely that they may progress to NASH [92]. 

In a recent work, MPV has been shown to be lower in women with MASLD associated with obesity and strictly correlated with early liver inflammatory response in NASH [98]. Specifically, platelet count and plateletcrit have been related to hepatic ballooning [98]. Duran-Bertran et al. presented a new predictive model by using MPV, ALT levels, and the presence of DM and MS to predict MASLD in obese women, displaying an area under the ROC curve of 0.84 [98].

Other emerging “platelet-derived” scores are the platelet to HDL-C ratio (PHR) and the NAFLD outcomes score (NOS).

The ratio of platelets/HDL-C has been showed to be significantly increased in patients with nascent metabolic syndrome, and it seems to be a valid biomarker of metabolic syndrome [99]; it may also be a biomarker for atherothrombotic risk in those subjects [99].

In a recent work by Calzadilla-Bertot et al., age, T2DM, levels of albumin, bilirubin, platelet count, and international normalized ratio were identified as independent predictors of liver-related events (LREs) [100]. Those factors were integrated into a comprehensive model termed the NAFLD Outcomes Score (NOS) [100]. It demonstrated excellent overall predictive accuracy; by using a cutoff value of ≥1.3, the NOS effectively stratified individuals into higher and lower risk categories for LREs [100]. Those surpassing this threshold exhibited significantly elevated risk (sub-HR 24.6, *p* < 0.001), with a 5-year cumulative incidence of 38%, compared to a mere 1.0% in those below the cutoff [100].

Assessment of predictive accuracy over time revealed exceptional performance of the NOS at both 5 and 10 years, as evidenced by time-dependent AUCs of 0.92 and 0.90 during derivation, and 0.80 and 0.82 during validation, respectively [100]. Notably, the NOS outperformed existing scoring systems such as the fibrosis-4 or NAFLD fibrosis score in predicting LREs at both 5 and 10 years (*p* < 0.001) [100].

In conclusion, patients with hepatic steatosis and NAFLD are characterized by higher values of PDW and MPV, while low platelet counts would be indicative of progression towards hepatic fibrosis.

## 5. Antiplatelet Therapy Effects on Liver in MASLD (NAFLD) Patients 

A growing body of evidence demonstrates how antiplatelet therapy can mitigate fibrosis development in NAFLD, thus identifying it as a potential antifibrotic agent [81,101,102,103,104]. In murine models, aspirin has been shown to exert an anti-inflammatory effect by limiting the activation of hepatic stellate cells through the inhibition of the pro-inflammatory enzyme COX-2 and by interfering with the PDGF signaling [81,104]. Fujita et al. performed a study on Fisher 344 male rats fed with a choline-deficient, l-amino acid-defined (CDAA) diet or a high-fat high-calorie (HF/HC) diet, and treated with or without the antiplatelet agents, aspirin, ticlopidine or cilostazol for 16 weeks [105]. All three antiplatelet drugs significantly decreased liver steatosis, inflammation, and fibrosis in the CDAA diet group [105]. Cilostazol was the most effective, and it also reduced liver steatosis in the HF/HC diet group [105]. Cilostazol exerts its beneficial effect against NAFLD by suppressing MAPK activation induced by oxidative stress and PDGF by intercepting signal transduction from a Akt to c-Raf [105]. Those findings are consistent with the results of a recent study conducted on human patients showing the association between the use of acetylsalicylic acid with reduced liver steatosis and fibrosis [106].

The association between acetylsalicylic acid and histological features of NAFLD has been further investigated in a prospective study conducted on a cohort of patients with histological diagnosis of NAFLD, followed by an extensive follow-up [50]. It has been shown that daily administration of acetylsalicylic acid is related to a 46% reduction in the risk of developing fibrosis compared to non-users. Notably, this effect is specific to the molecule itself and not to the class of drugs, as other NSAIDs, lacking antifibrotic action, do not reduce the incidence of advanced fibrosis [50]. 

A cross-sectional study analyzing data from the National Health and Nutrition Examination Survey III (NHANES III) specifically showed that the effect is linked to acetylsalicylic acid, unlike ibuprofen [106]. This study highlighted how aspirin use is significantly associated with lower fibrosis indices (e.g., FIB4, APRI), especially in individuals at risk of chronic liver diseases, with a consistent negative association across several chronic liver diseases (chronic viral hepatitis, suspected alcoholic liver disease, and NASH) [106].

The possibility of a correlation between regular aspirin intake and a reduction in the prevalence of NAFLD was demonstrated also by Shen and colleagues in a study of the general population in the United States involving over 11,000 adults [107]. Regular aspirin use, compared to not using it, was significantly related to a 38% lower probability of developing NAFLD [107]. The probability of developing NAFLD among men was less than half in those who reported occasional use of aspirin and two-thirds in those who used it regularly [107]. Aspirin use and NAFLD were not found to be correlated in women and younger participants, possibly because women have higher urinary excretion of aspirin and its metabolites than men, and younger subjects have higher aspirin clearance than older subjects [107]. Therefore, it can be argued that younger or female patients can need increased doses of aspirin to obtain a pharmacological effect against MASLD. However, the lack of information on exact doses of aspirin made it difficult to evaluate dose-dependent associations between aspirin and MASLD. Furthermore, MASLD diagnosis was based on ultrasound but was not confirmed by liver biopsy; then, some patients can have been misclassified as having or not having MASLD. According to the findings, taking aspirin regularly may lead to a lower risk of NAFLD, especially in men or older adults [107]. However, as a cross-sectional study, it was not possible to assess whether the use of aspirin could prevent the incidence or reduce the progression of NAFLD [107].

The use of aspirin was also proven to be related to relevant decreased liver fibrosis indexes in adult subjects with suspected chronic liver disease [106]. According to a systematic review and meta-analysis, there is a beneficial relationship between antiplatelet therapy and the prevalence of advanced liver fibrosis in MASLD patients [108]. 

In the work of Simon et al., longer duration of aspirin use was related to progressively reduced risk for incident advanced fibrosis, and this association was similar for any age and sex [50].

In a prospective work performed on subjects with bioptically proven NAFLD, daily use of aspirin was related to less severe histological characteristics of NAFLD and NASH and a decreased risk of progression towards advanced fibrosis [50]. Compared to non-regular use, daily use of aspirin has been associated with lower risk of NASH [odds ratio adjusted (aOR), 0.68; 95% confidence interval (CI), 0.37–0.89] and fibrosis (aOR, 0.54; 95% CI, 0.31–0.82) [50]. Daily consumers of aspirin had a significantly lower risk of developing advanced fibrosis than nonhabitual consumers [hazard ratio adjusted (aHR) [109], 0.63; 95% CI. 0.43–0.85] [50]. That correlation seemed to depend on duration, with the greatest beneficial effect observed with at least 4 years of aspirin use (aHR, 0.50; 95% CI, 0.35–0.73) [50].

Several molecular mechanisms have been suggested to explain the possible efficacy of antiplatelet therapy against NAFLD and its progression to NASH. Aspirin may stimulate NO and prostacyclin production through the production of endothelial NO synthase and vascular endothelial growth factor, thus inducing antioxidant activity [110]. Moreover, aspirin can inhibit the production of TNF-α, a relevant molecule involved in inflammation and liver fibrosis development [111]. Furthermore, aspirin reduces the expression of PDGF, thus decreasing hepatic inflammation, steatosis, and fibrosis [112]. Antiplatelet therapy can stimulate the insulin signaling pathway and improve IR by PDGF-induced activation of the mitogen-activated protein kinase pathway [113,114]. Eventually, aspirin-induced lipoxins in the liver regulate cytokine–chemokine axes in liver cells [115]. Malehmir et al. demonstrated that anti-GPIbα antibody treatment can decrease several cytokines and platelet-derived molecules [61]. Moreover, P2Y12 antagonist treatment, as well as depletion of functional GPIbα or lack of α-granules can decrease activation, accumulation, and adhesion of platelets to the liver endothelium, thus ameliorating liver damage and attenuating fibrosis development [61].

Furthermore, in the presence of hyperlipidemia and metabolic dysregulation, many of the biological substrates and conditions, such as IR and pro-inflammatory cytokines, are involved in the pathogenesis of both NAFLD and atherosclerosis [116]. Aspirin has been shown to improve both atherosclerosis and NAFLD simultaneously [117]; this mainly occurs through the following two pathways: by activating catabolic lipid metabolism (decreased lipid accumulation in HepG2 cells, aorta, and liver) and by reducing inflammatory response (decrease in NF-κB and TNF-α) [117].

## 6. Antiplatelet Therapy and Cancer

Hepatocellular carcinoma (HCC) incidence is increasing more rapidly than any cancer, with an age-adjusted annual increase in 3.8% and 2.8% in men and women in the U.S., respectively [118,119]. Several epidemiological studies confirmed the increasing incidence and risk of NAFLD-associated HCC [120,121]. The increasing rates of NAFLD-associated HCC are also demonstrated by changes in liver transplantation (LT) indications [122]. The European Liver Transplant Registry database from 2002 to 2016 showed that 8.4% of transplant patients were for NASH in 2016 (compared to 1.3% in 2002), 39% of whom had HCC [123]. 

Pharmaceutical inhibition of platelet function by using antiplatelet treatment have been previously shown to correlate with better outcome in cancer patients [124,125,126]. Therefore, the potential cancer chemo-preventive role of antiplatelet drugs in NAFLD/NASH patients, especially aspirin and other NSAIDs, represents an intriguing research area [127].

A recent meta-analysis conducted by the AISF (Associazione Italiana per lo Studio del Fegato) HCC Special Interest Group, which encompassed data from 20 studies, revealed compelling findings regarding antiplatelet therapy’s impact on HCC [128]. The analysis indicated that patients undergoing antiplatelet therapy exhibited a notable 40% reduction in the risk of HCC incidence [128]. Moreover, among individuals with HCC undergoing either curative or palliative treatment strategies, antiplatelet therapy was associated with a remarkable halving of the risk for all-cause mortality [128]. In this meta-analysis, patients with a clear indication for antiplatelet therapy were evaluated, but there was no specific evaluation for subgroups of patients nor for the NAFLD/NASH field specifically.

Another study analyzing prospective data of the National Institutes of Health—AARP Diet and Health Study cohort demonstrated that aspirin users had a statistically significant reduced risk of HCC incidence and mortality due to chronic liver disease (CLD) compared to those who did not use aspirin [129]; otherwise, users of non-aspirin nonsteroidal anti-inflammatory drugs (NSAIDs) had a reduced risk of mortality due to CLD but did not have lower risk of HCC incidence in comparison with those who did not use non-aspirin NSAIDs [129]. This observation could potentially stem from variations in the COX inhibitory effects between aspirin and NSAIDs. Aspirin, unlike other NSAIDs, exerts its effects by irreversibly inhibiting and modifying both isoforms of COX: the constitutive COX-1, prevalent in most normal tissues, and the inducible COX-2, which is highly expressed in response to various proinflammatory stimuli, including those implicated in hepatic carcinogenesis [129]; moreover, while aspirin irreversibly inhibits COX isoenzymes, NSAIDs do so reversibly, with transient effects that can reduce anti-fibrotic activity [129].

Lee et al. evaluated 35,898 cirrhotic patients dividing them into two groups (those treated with aspirin for at least 84 days vs. controls without treatment) [130]. Daily aspirin usage was found to be independently associated with a reduced risk of HCC, as evidenced by a three-year HR of 0.57 (95% CI 0.37–0.87; *p* = 0.0091) and a five-year HR of 0.63 (95% CI 0.45–0.88; *p* = 0.0072). This inverse correlation was notably linked to the duration of treatment, with distinct HR observed for varying treatment durations: 3–12 months (HR 0.88, 95% CI 0.58–1.34), 12–36 months (HR 0.56, 95% CI 0.31–0.99), and ≥36 months (HR 0.37, 95% CI 0.18–0.76) [130]. The overall mortality rates were significantly lower among individuals using aspirin compared to untreated controls, as evidenced by a three-year HR of 0.43 (95% CI 0.33–0.57) and a five-year HR of 0.51 (95% CI 0.42–0.63) [130]. There was no observed increase in the incidence of GI bleeding among daily aspirin users compared to untreated cirrhotic patients, regardless of whether they had a previous history of GI bleeding or not [130].

Furthermore, Choi et al. evaluated LT recipients with pre-transplant HCC and divided them into two groups based on the use of antiplatelet agents for >90 days or not [131]. The 5-year cumulative incidences of HCC recurrence and HCC-specific death were found to be comparable between the antiplatelet and non-antiplatelet groups [131]. The rates of all-cause and non-HCC deaths were also similar between the two groups [131]. 

A recent study compared the outcomes between 33,484 patients with NAFLD who continuously received a daily aspirin for 90 days or more, and 55,543 subjects who had not received antiplatelet therapy, by using Taiwan’s National Health Insurance Research Database [132]. 

The 10-year cumulative incidence of HCC in the treated group was significantly lower compared to that in the untreated group (0.25% [95% CI, 0.19–0.32%] vs. 0.67% [95% CI, 0.54–0.81%]; *p* < 0.001) [132]. Aspirin therapy was significantly related to a lower HCC risk (aHR 0.48 [95% CI, 0.37–0.63]; *p* < 0.001) [132]. In older patients (age > 55 years) with elevated serum ALT levels (considered high-risk patients), the 10-year cumulative incidence of HCC in the treated group was significantly lower compared to that in the untreated group (3.59% [95% CI, 2.99–4.19%] vs. 6.54% [95% CI, 5.65–7.42%]; *p* < 0.001) [132]. Moreover, HCC risk was significantly lower by using aspirin for ≥3 years (aHR 0.64 [95% CI, 0.44–0.91]; *p* = 0.013), in comparison with short-term use (<1 year) [132].

Tan et al. performed a metanalysis on 147,283 participants by including 19 studies evaluating patients with hepatitis B virus (HBV), hepatitis C virus (HCV), alcohol-related liver disease (ALD), or NASH that were administered at least one NSAID or antiplatelet therapy for a defined period of time and were followed for at least 6 months [133]. Aspirin usage was associated with a reduced risk of HCC incidence, with a HR of 0.51 (95% CI: 0.36–0.72). Additionally, aspirin was found to improve liver-related mortality, with an OR of 0.32 (95% CI: 0.15–0.70). However, it was also associated with a small increased risk of gastrointestinal bleeding events, with an OR of 1.32 (95% CI: 1.08–1.94) [133]. Regarding HCC recurrence following treatment, analysis of all aspirin and NSAID treatment collectively showed a decreased risk of HCC recurrence, with a HR of 0.80 (95% CI: 0.75–0.86) [133]. Through stratified analysis, it was observed that only the non-aspirin NSAID group exhibited a significant reduction in risk, with a HR of 0.73 (95% CI: 0.63–0.84) [133].

Therefore, there is evidence that the use of antiplatelet drugs (both aspirin and others) is correlated with a reduced incidence of HCC. Regarding the type of drug (aspirin vs. other NSAIDs) there are non-univocal data, in some cases conflicting [129,133], which deserve studies based on specific molecular mechanisms.

### Molecular Mechanisms

Antiplatelet drugs, in particular aspirin, have been investigated as cancer preventatives, particularly in the context of colorectal cancer [127,134]. A recent survey demonstrated that among subjects who had previously taken aspirin, 1.9% had taken it for cancer prevention [135]. Participants with a personal or family history of cancer were found to be more inclined to perceive aspirin as necessary for cancer prevention [135]. Concerns regarding the consumption of aspirin at higher doses and its associated side effects, particularly gastrointestinal bleeding, were prevalent among participants [135].

**Figure 3:**
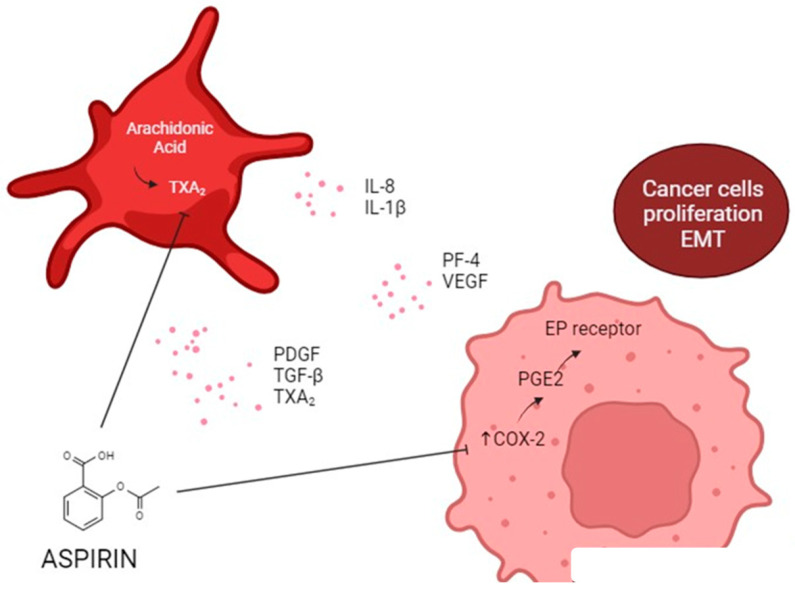
Summary figure on the anti-cancer mechanisms of antiplatelet therapy (aspirin). Aspirin can block COX-2 both at the platelet level and in tumor cells. At the platelet level, COX blockade reduces platelet activation and the release of mediators that may be involved in tumorigenesis (e.g., TGF-β, VEGF, TXA2). At the tumor cell level, COX-2 blockade appears to be able to reduce cell proliferation and epithelial–mesenchymal transition. Abbreviations: thromboxane A_2_, TXA_2_; interleukin, IL; platelet-factor-4, PF-4; vascular endothelial growth factor, VEGF; platelet-derived growth factor, PDGF; transforming growth factor β, TGF-β; cyclooxygenase, COX; prostaglandin E, PGE; prostaglandin E receptor, EP; epithelial–mesenchymal transition, EMT. Created in https://www.biorender.com/.

The beneficial effects of antiplatelet therapy for cancer can be attributed to the involvement of TX-dependent platelet activation and the COX-2-driven inflammatory response during the early stages of carcinogenesis [136,137] (Figure 3). Joharatnam-Hogan et al. measured urinary 11-dehydro-thromboxane B_2_ (U-TXM), a biomarker of in vivo platelet activation, in patients with breast, colorectal, gastro-esophageal, and prostate cancers after radical therapy [138]. An amount of 100 mg aspirin daily decreased U-TXM similarly across all tumor types; however, 300 mg of aspirin daily provided no additional suppression of U-TXM compared with 100 mg [138].

The beneficial effects of antiplatelet drugs against the development of HCC in patients with NAFLD can be divided into two main mechanisms. On the one hand, the slowdown in the progression towards liver fibrosis and NASH (as previously described), and on the other a possible direct effect of platelets and their mediators on carcinogenesis mechanisms.

A recent work by Ma et al. demonstrated that the inhibition of the P2Y12 receptor on platelets can promote tumor growth via CD40L in mice with NAFLD [139]; however, there are conflicting data on this mechanism in the literature. Indeed, in the context of MASLD and HCC, platelets exhibit a dual role. They can contribute to pro-hepatocarcinogenesis by promoting NASH pathology. However, they can also demonstrate anti-tumor activity against established tumors by stimulating CD8+ T cell responses [124,125,126,140,141].

Previous studies showed that platelets can enhance adaptive immunity [140,141], and activated platelets can release several molecules from α-granules, such as CD40L (Table 1). Several studies in the literature showed CD40L to exert a role in anti-cancer immunity, and platelets are the main source of circulating CD40L [142]. Platelet-derived CD40L is more greatly released in both NAFLD mouse models and patients with NASH [139]. Moreover, TGF-β released from platelets can impair T cell anti-tumor function [143].

In a recent study conducted by Vogt et al. in mice, it was demonstrated that intratumoral co-stimulation with CD40L-expressing dendritic cells (DC) led to a significant enhancement in vaccination efficacy when combined with murine alpha-fetoprotein (AFP)-transduced DC in pre-established HCC in vivo [145]. The combined therapy induced an early and robust Th1-shift in the tumor microenvironment, accompanied by increased tumor apoptosis. This led to synergistic tumor regression of HCC [145].

Different platelet inhibition strategies can affect platelet CD40L release differently, thus inducing opposite effects on HCC tumor growth in NAFLD [139]. Actually, Santilli et al. previously showed TX-dependent CD40L release in patients with T2DM, showing dose-dependent inhibition of CD40L circulating levels upon aspirin administration and after slow recovery after aspirin withdrawal [56].

Indeed, COX inhibition does not effectively block platelet CD40L release; this phenomenon is dependent on intracellular calcium levels and protein kinase C activation. However, it does not involve tyrosine kinases or the extracellular signal-regulated kinase (ERK) or p38 pathways. Additionally, COX enzymes are downstream of ERK and p38 signaling during platelet activation [148]. Therefore, P2Y12 inhibition-related beneficial effects on HCC development in NAFLD may not be found using COX or phosphodiesterase 3 (PDE3) inhibitors [139]. Blocking platelet aggregation while preserving CD40L release using non-P2Y12 inhibitors such as aspirin might be more appropriate for HCC patients with MASLD [61].

Recent data demonstrated an IL-12-dependent increase in CD40L production in bone marrow megakaryocytes in NAFLD models [139,144]. In particular, macrophages can induce an increase in IL-12 in the liver and CD40L production from bone marrow megakaryocytes [139]. A recent study has shown that the expansion of hepatic dendritic cells in NASH is triggered by heightened production of dendritic cell progenitors within the bone marrow, coupled with enhanced recruitment to the liver [149]. Moreover, megakaryocytes harbored in the lung can produce higher CD40L amount [139,149]. Intriguingly, the lung could influence liver tumor regulation in MASLD by modulating platelet function and CD40L release.

Furthermore, findings from a recent study involving obese patients suggested that in individuals with NAFLD, CD40L protein production is stimulated in megakaryocytes rather than directly in NAFLD or tumors. This suggests a mechanism involving the transfer of CD40L pre-mRNA or mRNA into platelets [150].

Eventually, CD40L is related to a strong CD8+ T cell response through CD40 licensing of dendritic cells [146]. Platelet-derived CD40L can increase CD8+ T cell activation [139] and their recruitment to liver in NAFLD [61]. Significantly, depletion of CD8+ T cells has been demonstrated to diminish the beneficial antiplatelet effects on HCC development in NAFLD. This suggests that the anti-HCC platelet function is at least partially mediated by CD8+ T cells [139,151].

## 7. Platelets and Cardiovascular Risk in MASLD (NAFLD) Patients

The disrupted blood clotting process observed in MASLD contributes to elevated rates of thrombotic events among affected individuals, thus amplifying the associated CV risk [16,152]. MASLD entails a multifaceted interplay between liver function and cardiometabolic risk factors, which collectively underpin conditions such as heart disease, arrhythmias, and CVD [153]. Modifications in platelet genetic expression and increased platelet aggregation correlate with higher CV risk in severely obese patients than in healthy subjects [154]. Moreover, surgical interventions for weight loss among obese individuals lead to distinct alterations in platelet gene expression patterns. These shifts in platelet transcripts appear to align with potential pathways implicated in reducing cardiometabolic risk subsequent to weight loss in MASLD [150]. An association has been noted between elevated MPV and an increased incidence of CV complications [155]. Conditions including stroke, thrombosis, and myocardial infarction have all been correlated with MPV levels [88]. In MASLD, high MPV values have been linked to an increased risk of atherosclerosis, suggesting its potential utility as a marker for CV events within this population [156]. A recent study indicated that individuals exhibiting an MPV exceeding 10.05, coupled with a NAFLD CV score surpassing 3.98, face heightened susceptibility to CVD, acute coronary syndrome, stroke, and related fatalities [157]. However, it is noteworthy that in cases of MASLD without accompanying metabolic risk factors such as obesity, hypertension, and diabetes mellitus, MPV may not serve as a reliable predictor of CV risk [158]. The presence of systemic inflammation is recognized as a contributing factor to CV events in individuals with MASLD. Alongside the pro-inflammatory characteristics of systemic platelets, elevated levels of vWF circulating in MASLD patients may offer insight into the linkage between low-grade systemic inflammation and heightened CV risk within this population [82]. Among obese adolescents diagnosed with MASLD, a notable correlation was observed between the APRI and carotid intima-media thickness, suggesting that elevated APRI scores could serve as a predictive indicator of worsening CV risk [159]. 

In the literature, there are no specific and extensive studies on the administration of antiplatelet therapy in subjects with hepatic steatosis or in patients included in the new definition of MASLD. Most studies involve the administration of antiplatelet therapy to patients with multiple cardiometabolic risk factors, and in particular to diabetic subjects. Aspirin monotherapy is commonly recommended for the secondary prevention of atherosclerotic cardiovascular disease events among both diabetic and non-diabetic patients [147]. Antiplatelet therapy should be considered for subjects with a high CV risk profile [160,161,162]. In a meta-analysis on primary prevention, aspirin use was demonstrated to reduce the risk of stroke in diabetic women and the risk of myocardial infarction in diabetic men [163]. 

In addition to its role in reducing CVD risk, aspirin has been found to lower fasting glucose, Hemoglobin A1c, and triglyceride levels in a dose-dependent manner among patients with T2DM, as evidenced by a previous meta-analysis [164]. Notably, T2DM patients may exhibit a diminished response to oral antiplatelet medications [165]. A meta-analysis demonstrated that triple antiplatelet therapy proved more effective in reducing the incidence of CVD-related morbidity, all-cause mortality, and revascularization compared to dual therapy among T2DM patients undergoing coronary stent implantation [166]. A recent meta-analysis has revealed that cilostazol exhibits greater efficacy and safety, particularly in terms of reduced bleeding risk, compared to aspirin and clopidogrel in secondary CVD prevention among patients with a history of ischemic stroke or transient ischemic attack [167]. Additionally, when used as an adjunct to dual antiplatelet therapy, cilostazol significantly lowers the risk of repeat revascularization and restenosis following coronary artery stent implantation, without impacting bleeding rates or stent thrombosis, according to meta-analytic findings [168]. Moreover, cilostazol has been shown to confer similar beneficial effects on peripheral vascular disease [169]. In patients with T2DM, cilostazol has demonstrated the ability to inhibit the progression of carotid intima-media thickness and albuminuria, reduce microalbuminuria, and prevent foot ulcers, as evidenced by studies [170].

## 8. Implications for Antiplatelet Therapy in Primary Prevention

The use of antiplatelet therapy in primary prevention has been scaled down in recent years on the basis of three large trials which highlighted a greater risk of bleeding complications compared to a benefit in terms of reduction of cardiovascular events [171,172,173]. Subsequent works and expert opinions reworked this type of evidence, thereby suggesting that in patients at higher cardiovascular risk (e.g., T2DM patients) the benefit of antiplatelet therapy exceeds the bleeding risk [174]. Nowadays, several international guidelines report different types of indications for the use of antiplatelet therapy in primary prevention, thus creating heterogeneity in prescription methods [175].

The recent “DCRM Multispecialty Practice Recommendations for the management of diabetes, cardiorenal, and metabolic diseases” argued that the use of aspirin is not generally recommended for patients without a history of ASCVD or other risk factors [147]. The Task Force suggests that prescribing aspirin for patients with two or more cardiovascular risk factors (such as advanced age, elevated non-HDL-C, elevated LDL-C, low HDL-C, diabetes, hypertension, CKD, cigarette smoking, family history of ASCVD, and elevated CAC score > 100) may be beneficial for individuals who are not at an elevated risk of bleeding [147].

Nowadays, the prescription of antiplatelet therapy in patients with MASLD is based mainly (solely) on the evaluation of cardio-metabolic comorbidities, with no reference to the degree of hepatic alteration; this is due to the lack of strong evidence and adequate studies.

A recent work randomized two thousand four hundred participants with NASH to a strategy with a polypill containing aspirin, atorvastatin, hydrochlorothiazide, and valsartan vs. a control group with the standard of care, with a 5-year follow-up (144). The adjusted relative risk of major cardiovascular events in the participants with NASH (0.35, 95% CI 0.17–0.74) was less than half that of the participants without NASH (0.73, 95% CI 0.49–1.00), although this difference did not reach statistical significance [176]. Moreover, the NAFLD patients exhibited a significant decrease in liver enzymes after 60 months of follow-up, with an intragroup reduction of −12.0 IU/L (95% CI −14.2 to −9.6) [176].

Furthermore, growing evidence in the literature refers to an inter-individual variability in response and efficacy to the use of antiplatelet therapy, particularly in some categories of subjects with specific comorbidities [177,178]. More specifically, antiplatelet therapy can be less effective in patients with a faster recovery of platelet COX-1 activity during the 24 h dosing interval [178]. 

A recent study by Simeone et al. showed that patients with accelerated COX-1 recovery are characterized by reduced platelet glycoprotein (GP)_Ibα_ shedding, and this can contribute to higher thrombopoietin (TPO) production and higher rates of newly formed PLT, escaping aspirin inhibition over 24 h [179]. Intriguingly, NAFLD and visceral obesity have been identified among clinical markers, together with younger age and higher TPO/GC ratio, as being predictive for the likelihood of faster COX-1 recovery and suboptimal aspirin response [179].

## 9. Conclusions

Growing data in the literature support future research on the use of an antiplatelet agent as a new treatment for MASLD. Nowadays, there are limited data from prospective studies on the effects of aspirin in patients with MASLD. Most studies available on the association between antiplatelet drugs and MASLD prevalence are observational. Prospective studies on randomized and controlled clinical trials are necessary to determine if antiplatelet therapy can prevent or delay the onset or progression of MASLD, whether this effect is restricted to certain population subgroups, and what type and dose range of antiplatelet agent is most effective. The use of antiplatelets drugs seems to have potential clinical implications for the prevention of HCC patients with MASLD, since platelets contribute to fibrosis progression and cancer development. Therefore, MASLD patients may require antiplatelet agents to prevent cardiovascular diseases, fibrosis progression, and cancer development.

## Figures and Tables

**Table 1:** Comparison between different evidence on the role of the CD40-CD40L pathway on carcinogenesis.

CD40 Pathway Anti-Tumor Activity	CD40 Pathway Pro-Tumor Activity
CD40L is related to a strong CD8+ T cells responses through CD40 licensing of dendritic cell [61,122,124,136,137,138,144].	Platelet-derived CD40L is greater released in both NAFLD mouse models and patients with NASH [139]
Co-stimulation with CD40L-expressing dendritic cells (DC) significantly improves vaccination by inducing an early and strong Th1-shift in the tumor environment as well as higher tumor apoptosis [145].	In NAFLD patients CD40L protein production is induced in megakaryocytes rather than NAFLD or tumors causing a transfer of CD40L pre-mRNA or mRNA into platelets [144].
Platelet-derived CD40L can increase CD8+ T cell activation and their recruitment to liver in NAFLD [61,139,146,147]	IL-12 dependent increase in CD40L production in bone marrow megakaryocytes in NAFLD models [139]
	Megakaryocytes harbored in lung can produce higher CD40L amount [139,147]
	Inhibition of the P2Y12 receptor on platelets can promote tumor growth via CD40L in mice with NAFLD [139]
